# Signal reachability facilitates characterization of probabilistic signaling networks

**DOI:** 10.1186/1471-2105-16-S17-S6

**Published:** 2015-12-07

**Authors:** Haitham Gabr, Tamer Kahveci

**Affiliations:** 1 Department of Computer and Information Science and Engineering, University of Florida, 32611, Gainesville, FL, USA

**Keywords:** probabilistic networks, signaling, reachability

## Abstract

**Background:**

Studying biological networks is of extreme importance in understanding cellular functions. These networks model interactions between molecules in each cell. A large volume of research has been done to uncover different characteristics of biological networks, such as large-scale organization, node centrality and network robustness. Nevertheless, the vast majority of research done in this area assume that biological networks have deterministic topologies. Biological interactions are however probabilistic events that may or may not appear at different cells or even in the same cell at different times.

**Results:**

In this paper, we present novel methods for characterizing probabilistic signaling networks. Our methods do this by computing the probability that a signal propagates successfully from receptor to reporter genes through interactions in the network. We characterize such networks with respect to (i) centrality of individual nodes, (ii) stability of the entire network, and (iii) important functions served by the network. We use these methods to characterize major *H. sapiens *signaling networks including Wnt, ErbB and MAPK.

## Background

Studying the structure and functions of individual biological molecules such as genes and proteins has led to major discoveries in molecular biology. However, in order to understand how cells function and respond to internal or external factors, it is crucial to extend our understanding beyond individual molecules to how these molecules collaborate through interactions. These interactions among molecules are often modeled as *biological networks*, in which nodes represent molecules and edges represent the interactions.

Signaling networks constitute one of the key classes of biological networks. These networks model how extracellular signals propagate inside the cell leading to designated responses. A signal starts at a receptor protein, typically located at the membrane. It propagates through a series of interactions between intermediate proteins and reaches to a reporter protein, typically a transcription factor. Analysis of these networks is of great importance, since their defects cause many disorders such as type-2 diabetes, Alzheimer, neurodegeneration, cancer, obesity, congenital malformations and osteoporosis [1-3].

A fundamental strategy in analyzing biological networks is to characterize their topological features computationally. There has been a plethora of studies to model a multitude of characteristics of biological networks. Among them, degree [4-6] or joint degree distribution [7], node centrality [8-10], network robustness [11,12] are just a few examples. We elaborate on the literature later in this section.

One inherent and key feature of biological networks that is often overlooked in the literature while characterizing them is that their topology is uncertain. Signaling networks share the same uncertainty. There are numerous reasons for such uncertainty. One of them arises from an inherent characteristic of the DNA replication process, that this process can initiate at many different locations on the chromosome with varying probabilities [13]. Furthermore, the set of initiation locations can vary across cells of even the same type. Recent studies have demonstrated that the replication timing as well as other epigenetic factors can alter the expression of the genes [14] and thus the probability of cis-acting and trans-acting interactions taking place in the cell. Therefore, even the most putative signaling networks are better off studied in an uncertain model.

Uncertainty of each interaction is often modeled as a probability value which shows the confidence in its presence [15]. These probability values can be obtained through some interaction databases, like STRING [16] and MINT [17], or through other methods of interaction data quality assessment such as linear regression of various factors including transcriptome and network topology [18,19]. In the rest of this paper, we call a network a *probabilistic network *if it contains at least one uncertain interaction. Otherwise, we call it a *deteriministic network*. We represent a probabilistic network as a graph *G *= (*V, E, P *), where *V *denotes the set of nodes (i.e., proteins), *E *denotes the set of edges (i.e., interactions), and *P *: *E *→[0, 1] denotes a function that returns the existence probability of each edge in *E*.

**Figure 1:**
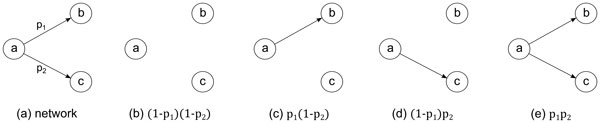
**An example probabilistic network with three nodes and two edges, and its four possible deterministic instances**. (a) The probabilistic network, where *p*_1 _and *p*_2 _are the probabilities of existence of the two edges. (b) through (e) are the 4 possible instances, where their probabilities are (1 − *p*_1_)(1 − *p*_2_), *p*_1_(1 − *p*_2_), (1 − *p_1_*)*p*_2 _and *p*_1_*p*_2 _respectively.

Most of the existing studies on characterizing networks are limited to deterministic network topologies. The main reason behind this limitation is that a probabilistic network summarizes a massive number of alternative topologies. More specifically, a *deterministic instance *of a probabilistic network represents a case where a certain subset of the edges in *E *exists and the rest do not. Thus, a probabilistic network *G *= (*V, E, P *) has 2|*E*| unique deterministic instances. Figure 1 illustrates this on a small probabilistic network with only two edges. Such exponential growth of the number of alternative topologies makes studying probabilistic networks an extremely challenging problem.

The vast majority of literature about characterization of biological networks considers them as deterministic networks, and ignores the probabilistic nature of their underlying topologies. The massive volume of research done in this area cannot be entirely covered in a few pages. We refer the interested readers to an extensive review on the topic [20]. In the following, we summarize some of the key recent studies.

**Studies on deterministic networks**. Jeong *et al*. [9] studied node centrality in protein interaction networks. They showed that the protein interaction network from *S. cerevisiae *follows a scale-free topology. They also showed that the chances that removal of a protein will prove lethal is proportional to the number of interactions the protein takes part in.

Yook *et al*. [4] presented methods for characterization of protein interaction networks. They characterized networks from four different datasets. First, they showed that both degrees and clustering indices of the nodes, along with the average cluster size, all follow power law distribution. Second, they studied the relation between network topology and both node functional and localization classes. Last, they studied the relations among functional and localization classes.

Jeong *et al*. [5] investigated the large-scale organization of metabolic networks from 43 different organisms. They showed that, in all studied organisms, the probability that a given substrate participates in *k *reactions (i.e. node degree) follows a power law distribution. Furthermore, when a randomly selected group of substrates are removed, the average distance among the remaining ones is not affected. This signifies a high level of robustness and low sensitivity to random perturbation.

Ravasz *et al*. [21] studied modularity in metabolic networks. They used the average clustering coefficient as a measure for modularity. They showed that metabolic networks follow a special hierarchical model where small modular subnetworks come together to form larger subnetworks, which in turn form larger subnetworks and so on. This model explains the scale free topology of metabolic networks, as well as their scaling clustering coefficients.

Kwon *et al*. [12] studied robustness in biological networks based on feedback dynamics. They showed that networks are likely to be more robust against perturbation if they have more positive feedback loops and fewer negative feedback loops. On the other hand, they also showed that nodes with large numbers of feedback loops are more essential to the network, and more lethal if mutated.

All these studies present valuable results about characteristics of various types of biological networks. However, they consider them having purely deterministic topologies. Hence, they fail to acknowledge and account for the probabilistic nature of biological events.

**Studies on probabilistic networks**. Relatively little research has been done for analysis and characterization of probabilistic biological networks. Network reliability of probabilistic networks has long been represented by special versions of the Tutte polynomial [22-24]. Such models represent the probability that an edge in the network fails. This facilitates the analysis of global characteristics of the network such as network connectivity. However, it does not facilitate the analysis of local characteristics, like node centrality.

Todor *et al*. [7] developed a novel method for characterizing the degree distribution of probabilistic biological networks. They used probability generating functions to model both degree distributions and joint degree distributions. They showed that power law and log-normal models are the best fit for degree distribution in probabilistic protein interaction networks. They also showed that, in such networks, nodes of high degrees are more likely to interact with nodes of low degrees. The method is specific to characterizing network degree distribution, with no results for network stability or individual node centrality.

We earlier developed an efficient method called *PReach *[25] for computing the reachability probability between sets of nodes in probabilistic signaling networks. We use this method in order to compute the reachability probability while implementing the methods described in this paper.

**Contributions**. In this paper, we test the hypothesis that the ability of signals to propagate between the proteins of a probabilistic signaling network determines the key characteristics of that network. The rationale behind this hypothesis is that through these signals, the proteins can regulate the expression levels of different genes and thus the cellular functions and responses. To test this hypothesis, we develop novel computational methods to characterize probabilistic signaling networks based on reachability probability. i.e., the probability that signals can successfully reach from its source nodes (i.e., receptors) to its target nodes (i.e. reporters). More specifically, we characterize signaling networks at three levels of granularity. At the lowest granularity level, we focus on individual proteins (i.e. nodes) of the underlying network. At the second level, we consider the entire network topology including all the proteins along with the interactions between them. At the highest level, we study the biological functions served by the network. Preliminary results for the three levels were published here [26]. We summarize each level next.

At the level of individual nodes, we investigate the centrality of each node in a probabilistic network. Node centrality is a metric that determines the relative importance of a node within the graph [9]. It is well defined in the scope of deterministic networks, with a number of variants including degree centrality and betweenness centrality [8]. However, it is not well defined in the scope of probabilistic networks. Here, we introduce a new node centrality measure that deals with the uncertainty of the network topology. This measure models the centrality of a node in terms of its contribution to the reachability probability between other nodes.

At the level of the entire network, we investigate the stability of the network with respect to the external factors that alter its topology or interaction probabilities on the ability of the network to carry out its functions. We say that a network is stable if small changes to its topology or edge probabilities do not cause massive changes in the probability that signals initiated at source nodes reach to target nodes. In other words, stability means that the network can continue to operate normally after such perturbations. We develop a new method for measuring stability of probabilistic signaling networks.

At the level of the biological functions we explore the set of functions that a given probabilistic signaling network performs. To do this, we use the Gene Ontology (GO) [27] term annotations of the source and target nodes of the given network. We develop two methods to model two orthogonal characteristics. The first one finds the most popular GO terms (i.e. the GO terms that are enriched by the most reachable target nodes). The second one finds which functions can be initiated with the highest probability (i.e., most reachable GO terms). Collectively, these two methods explain the prevalent functions that are carried out by a given signaling network through propagating signals from receptors to reporters.

## Results and discussion

In this section, we present experimental results for characterization of probabilistic signaling networks. We used the *Homo sapiens *signaling networks taken from KEGG [28] in our experiments. Among those, we used the largest ones (i.e., networks with more than 50 edges), which are ErbB, MAPK and Wnt. We obtained the sources and targets of each signaling network based on the hierarchical organization of its proteins [29]. We set the genes at the top of the hierarchy as the source nodes and the ones at the bottom as the target. We extracted the confidence scores for each interaction from STRING [16] and used them as edge probabilities. STRING computes the confidence values by benchmarking groups of associations against the KEGG functional classification scheme, which is manually curated. STRING has confidence values in the [1, 1000] interval, where 1000 indicates 100% confidence. We normalized this number to the[0, 1] interval for each interaction by dividing by 1000.

### Node centrality in probabilistic networks

In this section, we present experimental results for measuring probabilistic node centrality in probabilistic signaling networks. As explained later in the Methods section, We measured the probabilistic centrality value for all proteins in ErbB, MAPK and Wnt. The first question we need to answer at this point is whether probabilistic networks yield different centrality values than deterministic ones. If yes, what is the significance of the difference? To answer these questions, we compared our results with the betweenness centrality of each node in the underlying deterministic topology, where all edges are certain. We used the betweenness centrality for comparison as it is used frequently in the literature[30-32]. Also, it is the closest centrality measure to ours in terms of the biological meaning of centrality. We ranked the proteins according to both centrality values separately. We then measured the *disagreement *between the two rankings as follows. For each protein *x*, we counted the number of proteins whose position relative to *x *in one of the ranking disagree with the other. In other words, a protein *y *was counted if it is more central in the deterministic centrality ranking and less central in the probabilistic one, or vice versa. We normalized the resulting number to the[0, 1] interval by dividing it by the total number of proteins in the network.

**Figure 2:**
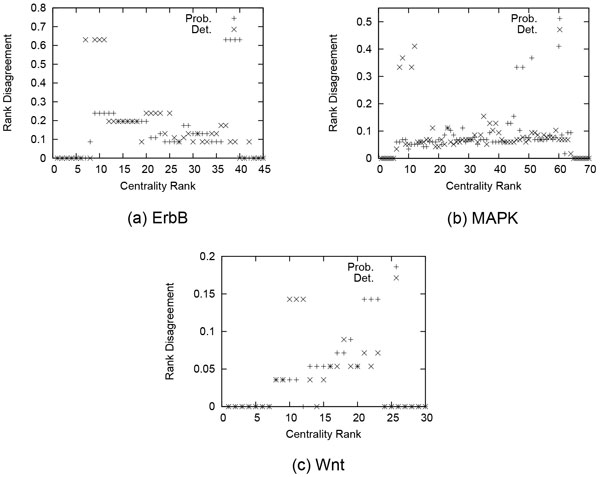
**Centrality rank disagreement of deterministic betweenness centrality (Det**.) and probabilistic centrality (Prob.) in (a) ErbB, (b) MPAK and (c) Wnt.

Figure 2 shows the disagreement value of all proteins when they are ranked according to probabilistic and deterministic centrality measures. The results demonstrate that both centrality measures completely agree on the proteins of the highest as well as the lowest centrality. The figure also shows severe disagreement for some of the proteins. We observe that these proteins are ranked highly based on the deterministic centrality measure. On the other hand, they are ranked at the low end by our method. In ErbB for instance, the first disagreeing rank is the seventh. Our method assigns this rank to ErbB3, which is a member of the ErbB family of receptor tyrosine kinases whose signaling mechanics regulate cell proliferation, differentiation, motility, and survival. On the other hand, the seventh rank in deterministic betweenness centrality is assigned to MAP2K7, which is not a member in the ErbB family and not as highly important as ErbB3 to the ErbB signaling process. This suggests that the probabilistic centrality measure is more suitable for probabilistic networks, because it is more likely to accurately point out highly central nodes. This is important because we are usually interested in identifying the most important proteins, so errors in identifying these are especially costly. The last observation we make is that the range of rank disagreement in ErbB is relatively larger than that of MAPK and much larger than that of Wnt. The little disagreement between the two rankings in case of Wnt means that we can use the deterministic measure with a little loss of information. This indicates that edge probabilities have a small role towards node centrality in Wnt, while the underlying deterministic topology has a more dominant role. On the other hand, the higher disagreement in case of ErbB means a large loss of information if we use the deterministic measure. This indicates that edge probabilities have a dominant role towards node centrality in ErbB, compared to the role of the underlying deterministic topology.

### Assessment of network stability

In this section, we evaluate the stability of probabilistic signaling networks ErbB, MAPK and Wnt using our method (see the Methods section). We measured network stability in terms of its reaction to random perturbation. The more a network maintains its signal reachability levels under perturbation, the more it is considered stable. On the other hand, if reachability levels drop dramatically in the event of perturbation, then it is considered unstable. We measured stability under two possible network perturbation models: alterations applied to the interaction probabilities and modifications applied to the network topology. We do not present results for MAPK in the topology perturbation experiment as measuring reachability probabilities in such a large number of randomly perturbed topologies takes more time than feasible in that experiment.

**Figure 3:**
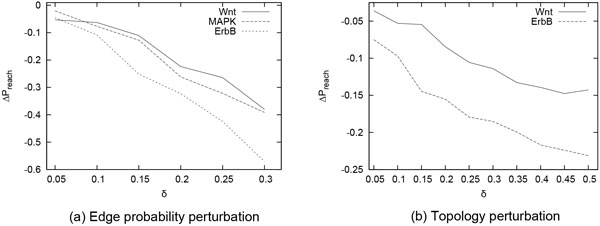
**Effect of random perturbations applied to the network on the reachability probability as a measure of network stability**. (a) Edge probability perturbation: average change in reachability probability (Δ*P_reach_*) when each interaction probability p is altered to a random value in the window *p *± *δ *∩[0, 1]. (b) Topology perturbation: average change in reachability probability (Δ*P_reach _*) when *δ *× |*E*| randomly selected edges are shuffled.

Figure 3 shows the results. We observe that, for both perturbation models, reachability probability monotonically decreases with the increase in perturbation. This observation implies that both the original topology and the original edge probabilities of the network constitute a local optimum for ensuring the signals travel from source to target nodes with a high chance. This is because random perturbations not only alter the reachability probability, but they also always tend to reduce this value. This is an extremely important observation as it can help in improving the accuracy of network construction algorithms. The drop in reachability probability varies from one network to another. This implies that different networks show different levels of sensitivity to random perturbations. ErbB showed largest drops in reachability probability, while Wnt was the most stable of the three. This signifies that functions served by the Wnt signaling network are more stable and can overcome random changes, while those served by ErbB are more prone to disruption under such changes. It is notable that this ordering holds for both topology perturbation and edge probability perturbation. This suggests that a network is more likely to be stable (or unstable) with respect to both perturbation models together.

### Characterizing network functions

In this section, we characterize the important functions performed by probabilistic signaling networks based on their enrichment and reachability, as explained in the Methods section. We use ErbB, MAPK and Wnt networks in our experiments.

**Figure 4:**
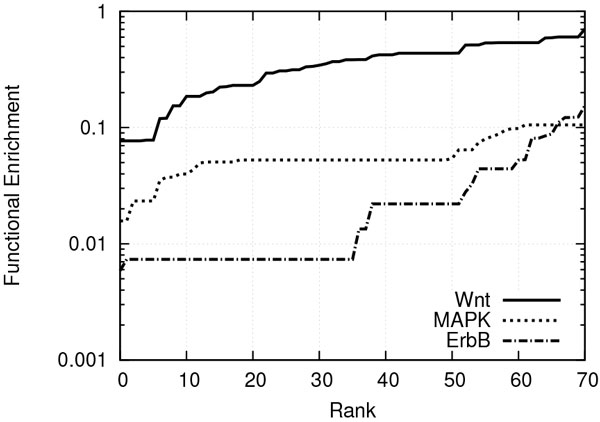
**Functional enrichment values in ascending order, for the top 70 terms in ErbB, MAPK and Wnt**.

We first measured the enrichment of the GO terms among the most reachable target nodes. We considered the terms with an enrichment value of 0.1 or lower as highly enriched. Figure 4 shows the enrichment values of the top 70 terms of each network. ErbB has 66 highly enriched terms out of 368 terms annotating the network proteins (18%), among which are asparagine metabolism (GO:0006528) and neuro-transmitter biosynthesis (GO:0042136). MAPK has 61 highly enriched terms out of 353 terms annotating the network proteins (17%), among which are optic vesicle formation (GO:0003403) and mechanoreceptor differentiation (GO:0042490). Wnt has only 6 highly enriched terms out of 295 terms annotating the network proteins (only 2%), among which are shmoo orientation (GO:0000753) and protein polyubiquitination (GO:0000209). The much lower number of enriched terms suggests that Wnt is more specific than ErbB and MAPK in terms of the functions it performs.

**Figure 5:**
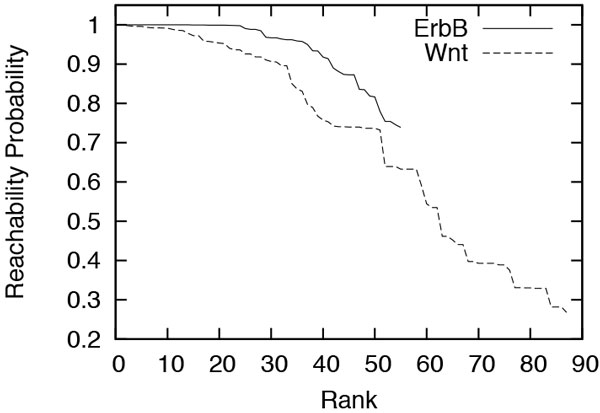
**Reachability probability of the GO terms of source and target proteins in ErbB and Wnt, in descending order**.

Next, we measured the reachability probability of the individual GO terms for the source and target proteins of each of ErbB and Wnt. Figure 5 plots these reachability probability values in descending order. We observe from the figure that Wnt has fewer GO terms annotating both its source and target proteins as compared to ErbB. However, it also shows that these fewer terms of Wnt are supported by higher reachability probability than all those of ErbB. This indicates that ErbB serves a broad spectrum of functions, while Wnt serves a more specific group of functions with stronger support. These results are consistent with our observations from Figure 4.

**Figure 6:**
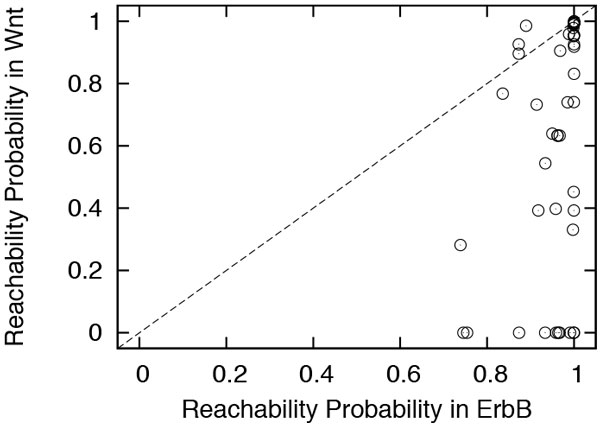
**The reachability probability of the common GO terms in ErbB and Wnt**.

One question that follows from Figure 5 is: Are the most reachable functions in one network specific to that network? In other words, can the two networks substitute each other for their most significant functions? We focused on ErbB and Wnt to seek and answer to this question. ErbB and Wnt share 48 common terms. For each of these common terms, we computed its reachability probability in both networks. Figure 6 shows the results. The majority of points (i.e., terms) are well below the diagonal. This observation supports the previous conclusion that ErbB serves with high probability a broader range of functions. The figure also shows relatively little overlap between the two networks with regard to the highly reachable terms. The few common highest reachable terms in both networks represent some important biological processes, such as protein polyubiquitination (GO:0000209), juvenile hormone metabolism (GO:0006716) and very-long-chain fatty acid metabolism (GO:0000038).

**Figure 7:**
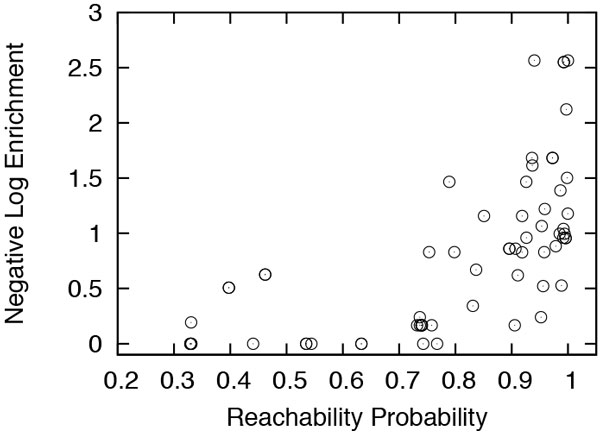
**Reachability probability of the GO terms in Wnt, versus the negative logarithm of their functional enrichment value**.

Next, we explore the possibility of a correlation between the reachability probability of the GO terms and their enrichment. Figure 7 shows the results for Wnt. From the figure we observe that terms with low reachability probabilities exhibit low enrichment levels. This implies that terms of low reachability represent functions that are not significantly served by the network. On the other hand, terms with high reachability probability are spread over a wide spectrum of enrichment values. The reason behind this is that highly reachable genes are annotated by both high and low-enrichment terms. Both kinds of terms inherit their high reachability values from the genes they annotate. Therefore, some terms with low enrichment levels happen to exhibit high reachability probabilities. Hence, the general conclusion we draw from the figure is that a high reachability probability is necessary, but not sufficient, for a function to be significantly served by the network.

**Figure 8:**
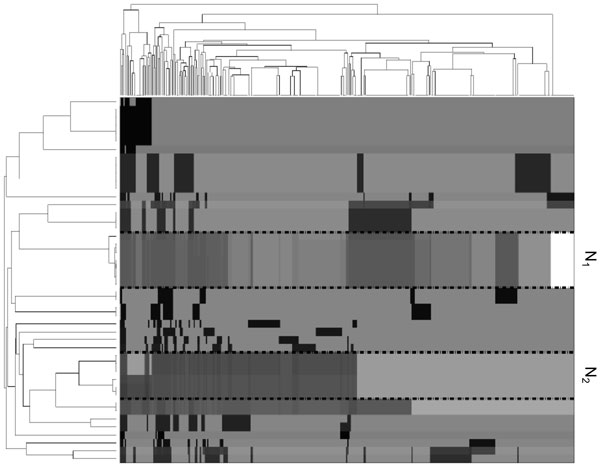
**A heat map of reachability probabilities from all proteins (rows) to all GO terms (columns) in ErbB network**. Each point at row *i *and column *j *represents the reachability probability from the *i^th ^*protein to the *j*^th ^GO term. Darker color indicates higher probability. Results are hierarchically clustered for identification of similar groups of proteins.

Next, we clustered similar proteins based on the reachability of their functional annotations. We measured the reachability probability from all proteins to all GO terms in the network. A target GO term denotes all target proteins annotated by this term. We performed hierarchical clustering [33] on the proteins based on their reachability probabilities to all GO terms. We plotted a heat map of these clustered reachability values. Figure 8 shows the results for ErbB. From the figure, we observe homogeneous groups of proteins such as the clusters labeled N1 and N2. For instance, cluster N1 consists of the proteins encoded by EGF, EGFR, TGFA, AREG, EREG, HBEGF and BTC, which are all members of the epidermal growth factor family of ligands and receptors. This family has an important role in cell growth and proliferation, tissue turnover and wound healing. Overexpression or mutation of its members is linked to a number of cancer types [34-37].

**Figure 9:**
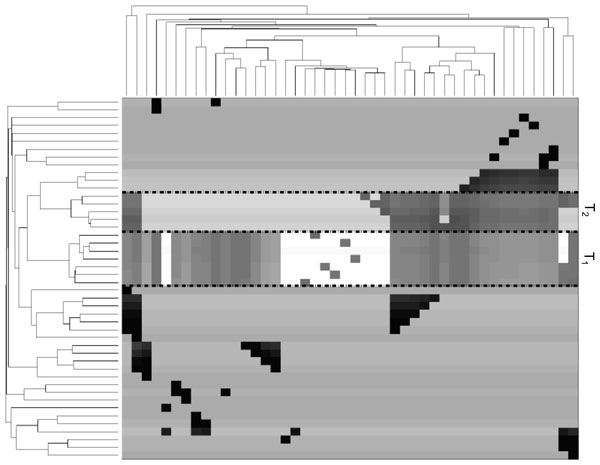
**A heat map of reachability probabilities between all pairs of proteins in ErbB network**. Rows are all proteins as sources, columns are all GO terms as targets. Darker color indicates higher probability. Results are hierarchically clustered for identification of similar groups of proteins.

It is worth noting that functional annotations of the network proteins represent extra information. It depends on the presence of an external ontology that is not necessarily available for all proteins in the subject network. Therefore, we pose the following question: does this grouping depend on the knowledge of the functional annotations of the network proteins? In other words, can we gain knowledge of such grouping without prior knowledge of such annotations? To answer this question we performed an experiment that disregards the knowledge of functional annotations. More specifically, we measured the reachability probability between all pairs of proteins in the network. We performed hierarchical clustering on the proteins based on their reachability probabilities to other proteins. We plotted a heat map of these clustered reachability values. Figure 9 shows the results for ErbB. We observed clusters of proteins that are almost identical to the ones in Figure 8. For instance, groups *N_1 _*and *N*_2 _in Figure 8 are almost totally identical to groups *T*_1 _and *T*_2 _respectively in Figure 9. These groups are detailed in Table 1. This observation shows that knowledge of functional annotations is not necessary for obtaining the described grouping.

**Table 1:** Details of gene groups marked in Figure 9 and Figure 8.

N1: EGF, EGFR, TGFA, AREG, EREG, HBEGF, BTC	T1: EGF, EGFR, TGFA, AREG, EREG, HBEGF, BTC
N2: SHC2, GRB2, ERBB3, PIK3R5, AKT3, GAB1	T2: SHC2, GRB2, ERBB3, NRG1, NRG2

## Conclusion

We developed a comprehensive set of methods for characterizing probabilistic signaling networks on three levels. First, we developed a method for measuring node centrality, based on the node's contribution towards the probability of signal reachability through the network. Second, we developed methods for characterizing the level of stability of the entire network, based on the amount of change in reachability probability when perturbing either the network topology or interaction probabilities. Last, we developed methods for characterizing the functional terms in the network, based on the enrichment and reachability of these terms.

We presented novel results by applying these methods to the ErbB, MAPK and Wnt signaling networks of *H. sapiens *obtained from KEGG. For node centrality, our results showed that the novel centrality measure we present here is more suitable for probabilistic signaling networks. We also showed that node centrality is dominated by the network topology in Wnt, as opposed to being more dominated by edge probabilities in ErbB. For network stability, Our results showed that the original topology and edge probabilities serve each network better than any randomly perturbed version of them. We also showed that Wnt showed the highest level of stability against random perturbations, while ErbB showed the lowest level of stability. Finally, for the functional terms, our enrichment and reachability results both showed that Wnt is a lot more specific than both ErbB and MAPK in terms of the functions it serves. Results also showed a little overlap between ErbB and Wnt in terms of the highly reachable functions. We also showed functional terms tend to be consistent in terms of both enrichment and reachability. Last, we showed that functionally-related groups of proteins can be identified by clustering of their reachability probabilities towards different functional terms, as well as towards other proteins.

This paper makes a significant contribution over the existing literature. First, it extends our understanding of biological networks from the simple deterministic topology to probabilistic. Second, we demonstrate that signaling networks can be computationally characterized in terms of the reachability of signals from receptors to reporters.

## Methods

In this section we present the novel methods we developed to arrive at the results explained above.We characterize probabilistic signaling networks based on the probability that signals can travel in the network, particularly from receptors to reporters. First, we present a method for measuring node centrality. Then, we present methods for evaluating the stability of a network with respect to random perturbation. Last, we present methods for characterizing biological functions served by the network based on their functional enrichments and reachability probabilities.

Throughout the rest of this paper, we use the following notation. We denote the set of nodes (i.e., proteins) by *V *and the set of directed edges (i.e., interactions) by *E*. We denote an edge probability function by *P *: *E *→ [0, 1], which returns the existence probability of each edge *e *∈ *E*. Consider a probabilistic signaling network *G *= (*V,E, P*), a set of source nodes *S *⊆ *V *, and a set of target nodes *T *⊆ *V *. We denote the reachability probability from *s *∈ *V *to *t *∈ *V *by *P_reach_*(*G, s, t*), which returns the probability of a signal reaching successfully from *s *to *t *in *G*.

### Computing reachability probability

We use *PReach *[25] for measuring the signal reachability probability between receptors and reporters in probabilistic signaling networks. Let *U *= {1*, . . . , n*}, where *n *= |*E*|. Let Θ be a subset of *U*. Let *S*_1_*, . . . , S_k _*be *k *different subsets of Θ. Let *X *and *Y *be two sets of *n *variables, where *X *= {*x*_1_*, . . . , x_n_*} and *Y *= {*y*_1_*, . . . , y_n_*}. Let $x_{S_{i}}=\prod_{j\in S_{i}}x_{j}$ and $y_{S_{i}}=\prod_{j\in S_{i}}y_{j}$, where *i *∈ {1*, . . . , k*}. Let *x*^∗ ^and *y*^∗ ^be two free variables. Let *a*_1_*, . . . , a_k_, b *and *c *be real numbers. PReach defines an ***xy*-*polynomial ***over Θ as $F=\sum_{i=1}^{k}a_{i}x_{S_{i}}y\Theta \backslash S_{i}+bx^{*}+cy^{*}$.

PReach associates every edge *e_j _*∈ *E *with a variable *x_j _*∈ *X *and a variable *y_j _*∈ *Y *, where *j *∈ *U. x_j _*designates the case where *e_j _*is present, while *y_j _*designates the case where *e_j _*is absent. Each of the non-free terms $a_{i}x_{S_{i}}y_{\Theta }\backslash S_{i}$ represents a combination where *e_j _*is present ∀*j *∈ *S_i _*and absent ∀*_j _*∈ Θ \ *S_i_. a_i _*is the probability of this specific combination. The free variable *x*^∗ ^designates the case where *T *is reachable from *S*, and *b *is its probability. Inversely, The free variable *y*^∗ ^designates the case where *T *is unreachable from *S*, and *c *is its probability.

Let *p_i _*= *P*(*e_i_*) and *q_i _*= 1 − *p_i_*. PReach proceeds by associating every edge *e_i _*∈ *E *with a binomial *p_i_x_i_*+*q_i_y_i_*. It then proceeds by multiplying these binomials into a growing *xy*-polynomial. After every mulitiplication, PReach checks the polynomial for non-free terms that can be *collapsed *into one of the two free terms. For any of the non-free terms *a_i_xS_i_y*_Θ_\*S_i _*, if the edge set associated with *S_i _*contains a path from *S *to *T *, the term is replaced by *a_i_x*^∗^. If the edge set associated with Θ \ *S_i _*contains a cut between *S *and *T *, the term is replaced by *a_i_y*^∗^. Any later multiplication of a new term *p_i_x_i _*with *bx*^∗ ^results in *bp_i_x*^∗^. Similarly, (*p_i_x_i_*)(*cy*^∗^) = *cp_i_y*^∗^, (*q_i_y_i_*)(*bx*^∗^) = *bq_i_x*^∗^, and (*q_i_y_i_*)(*cy*^∗^) = *cq_i_y*^∗^. Therefore, the size of the *xy*- polynomial avoids growing in an exponential rate.

### Characterizing node centrality

The smallest building blocks of a probabilistic signaling network are the individual nodes that make up the network. Therefore, as a first step in characterizing these networks, we focus on the roles of individual nodes in how signaling networks function. To do that, we develop a new model to explain the centrality of individual nodes.

Our method mimics the *betweenness *centrality measure. Traditionally, this measure has been frequently used for deterministic networks. In such studies, it considers a node *x *to be between nodes *y *and *z *if *x *is on the shortest path from *y *to *z*. These studies however have two major flaws. First, a probabilistic network can yield many alternative deterministic network topologies. As a result, different sets of nodes can be between *y *and *z *for different deterministic topologies. Thus, it is not certain whether *x *is in that set. Second, there is no guarantee that a signal traveling from *y *to *z *will always choose the shortest path. Thus, limiting betweenness to only the shortest paths is unrealistic.

We develop a new method for measuring node centrality in a probabilistic network based on reachability probability. We consider a node as highly central in a probabilistic network if a signal traveling from a source node to a target node visits that node with a high probability. Based on this, we measure the node centrality as the expected number of source-target pairs whose connectedness relies on the presence of the subject node. We explain this in detail next.

Given a node *v *∈ *V *and a source-target pair (*s, t*), we call *v *an essential node for (*s, t*) if the removal of *v *from the network disconnects *s *and *t*. Given a node *v*, for each source-target pair (*s, t*), we want to measure the probability of *v *being essential for (*s, t*). To do this, we first measure the probability of a signal propagating successfully from *s *to *t *given the existence of *v*. This value is denoted by *P_reach_*(*G, s, t*). We then measure that probability in the absence of *v*. To do this, we construct a modified network *G*′ by removing *v *and all its incoming and outgoing edges. We then compute the reachability probability *P_reach _*(*G′, s, t*). The difference between the first and the second probability values represents the probability of a signal having to pass through *v *in order to reach from *s *to *t*. Therefore, given these two probability values, we calculate the probability of *v *being an essential node to (*s, t*) as *Cv*(*G, s, t*) = *P_reach_*(*G, s, t*) − *P_reach_*(*G′, s, t)*.

For a given node v, given the value of *C_v _*(*G, s, t*), ∀_s _∈ *S, t *∈ *T *, we compute the centrality of *v *as the average number of (*s, t*) pairs for which *v *is essential. To do this, we consider the random variable *Xv *that follows Poisson Binomial distribution with parameters *C_v_*(*G, s, t*), ∀*s, t*. Thus, the expected number of (*s, t*) pairs for which *v *is essential becomes equivalent to the expected value $E\left[X_{v}\right]=\sum_{s\in S,t\in T}C_{v}\left(G,s,t\right)$.

Computing the centrality of a node involves computing reachability two times: before and after removing the node. Therefore, the time complexity is the same as that of PReach O(2^|*E*|^). However, this is a theoretical upper bound, as PReach avoids the exponential growth in practice[25].

### Characterizing network stability

In the previous section, we characterized individual nodes in probabilistic signaling networks. Here, we expand our model to characterize the entire network. More specifically, we develop a method for evaluating stability of probabilistic signaling networks. Briefly, we say that a network is stable if the probability that a signal travels successfully from source to target nodes in that network does not change greatly when a small amount of random perturbations are applied to that network. We consider two types of perturbations: (i) alteration of edge probabilities and (ii) modification of network topology. We describe a parametric model for each of them later in this section.

Consider the given network *G *= (*V, E, P *) and the sets of source and target nodes *S *and *T *. Let us denote the network obtained after perturbing *G *with *G^δ ^*. Given a source node *s *∈ *S *and a target node *t *∈ *T *, the difference *P_reach_*(G^δ ^, s, t)−*P_reach_*(*G, s, t*) indicates the change in the reachability probability from *s *to *t *after the network is perturbed. We compute the stability of *G *with respect to *G^δ ^*in terms of the average of this difference over all possible pairs of *s *∈ *S *and *t *∈ *T *(i.e., $\frac{1}{\left|S\right|\times \left|T\right|}\sum_{s\in S,t\in T}\left(P_{reach}\left(G^{\delta },s,t\right)-P_{reach}\left(G,s,t\right)\right)$. A large magnitude for this value indicates that *G *is unstable. The sign of this value shows the direction of instability. A negative sign indicates a drop in reachability and thus the cell getting unresponsive to external signals. A positive sign indicates a rise in reachability and thus the cell getting over-sensitive to such signals.

Next, we describe in detail how we model perturbation of a network *G *given a perturbation parameter *δ *to obtain a perturbed network *G^δ ^*.

• *Perturbation of edge probabilities*. In this case, the parameter *δ *denotes the maximum change in edge probabilities. We define a perturbed edge probability function *P ^δ ^*→[0, 1] that, for each edge *e *∈ *E*, returns a value drawn uniformly at random from the range *P *(*e*) ± *δ *∩[0, 1]. We construct *G^δ ^*= (*V, E, P ^δ ^*).

• *Perturbation of network topology*. In this case, we inflict topology perturbation by degree-preserving edge shuffling for a fraction of the edges. Each shuffling operation randomly picks a pair of edges (*u*_1_, *v*_1_) and (*u*_2_, *v*_2_). It then replaces these edges with (*u*_1_, *v*_2_) and (*u*_2_, *v*_1_) and randomly assigns each of the old edge probabilities to the new ones. The parameter *δ *denotes the fraction of edges to be shuffled. We construct *G^δ ^*= (*V, E^δ ^, P *), where *E^δ ^*is obtained from *E *by randomly shuffling a fraction *δ *of the edges.

Similar to computing node centrality, characterizing network stability involves computing reachability two times: before and after introducing the perturbation. The time complexity is the same as that of PReach *O*(2^|*E*|^). Again, this is a theoretical upper bound, as PReach avoids the exponential growth in practice[25].

### Characterizing network functions

Each signaling network is responsible for carrying out various functions. In this section, we develop a method to mathematically characterize the biological functions that can be realized by a given probabilistic signaling network. We use the GO terms associated with the target genes to denote the set of possible functions of the underlying signaling network. The GO database organizes terms in a hierarchy of "is-a" and "part-of" relationships, such that the highest level is the most generic. We ignore the terms in the top five levels of the hierarchy in our analysis. Note that these terms are commonly ignored as they are generic and commonly assigned to a large number of genes [15].

A target gene's ability to perform the functions it is annotated with is affected by the extracellular signals which reach to that gene. Following from this observation, we conjecture that a network is more likely to regulate a function if the set of target genes annotated with that function are reachable with higher probability than the other genes. More specifically, we model two different characteristics of the functional annotations of a probabilistic signaling network in terms of reachability of the nodes of that network. Namely, these are the enrichment and the reachability of the annotations. We elaborate on them next.

**Enrichment of functional annotations**. Consider the given network *G *= (*V, E, P *) and the sets of source and target nodes *S *and *T *. For each target node *t *∈ *T *, we compute the reachability probability from at least one of the source nodes in *S *to *t*. The resulting reachability probabilities provide a ranking of the target node in *T *. We then consider the set A of all GO terms in *G*. For each term *a *∈ *A*, given a parameter *d *∈ {1, 2, . . . |*T*|}, we consider the set *T_d _*⊂ *T *as the set containing only the top d target nodes in *T *with the highest reachability probability. We calculate the enrichment value for *a *in *T_d _*as follows. Let *N *be the number of all targets in *T *annotated with *a, n *be the number of targets annotated with *a *in *T_d_*. We compute the enrichment value of a as *P *(*X *≥ *n*||*T *|, *d, N *) where *X *is a random variable under a hyper-geometric distribution with these parameters. We try all possible values of *d *∈ 1, . . . , |*T *| and select the best enrichment value. In other words, the enrichment value shows the probability that a random subset of *T *of size *d *contains at least n nodes annotated with *a*. The lower the enrichment value is, the more significant the term *a *is.

**Reachability of functional annotations**. Consider the given network *G *= (*V, E, P *) and the sets of source and target nodes *S *and *T *. Also consider the set *A *of all GO terms in the network. For each *a *∈ *A*, we construct the two sets *S_a _*⊂ *S *and *T_a _*⊂ *T *. Here, *S_a _*and *T_a _*denote the set of source and target nodes that are annotated by *a *respectively. We measure the reachability probability of *a *as the reachability probability from any node in *S_a _*to any node in *T_a_*. We rank the terms in A according to their reachability probabilities. Higher reachability probability of a term means that the paths available for the associated function contains more reliable interactions and/or more redundant paths. Therefore, we expect that the functional terms with higher reachability probability play more critical roles than others within that network.

The time complexity of both methods is dominated by that of calculating reachability probability. Therefore, the time complexity is the same as that of PReach*O*(2^|*E*|^). Again, this is a theoretical upper bound, as PReach avoids the exponential growth in practice[25].

## Data access

All data, scripts and results are available at http://bioinformatics.cise.ufl.edu/PReach/characterization.htm

## Competing interests

The authors declare that they have no competing interests.

## Authors' contributions

HG participated in methods design and implementation, data set collection, experiments design and implementation, analysis of the results and writing of the paper. TK participated in methods and experiments design, analysis of the results and writing of the paper. All authors read and approved the final manuscript.

## Declarations

Publication of this article was funded by NSF under grant CCF-1251599.

This article has been published as part of *BMC Bioinformatics *Volume 16 Supplement 17, 2015: Selected articles from the Fourth IEEE International Conference on Computational Advances in Bio and medical Sciences (ICCABS 2014): Bioinformatics. The full contents of the supplement are available online at http://www.biomedcentral.com/bmcbioinformatics/supplements/16/S17.
